# l-amino acids affect the hydrogenase activity and growth of *Ralstonia eutropha* H16

**DOI:** 10.1186/s13568-023-01535-w

**Published:** 2023-03-17

**Authors:** Meri Iskandaryan, Syuzanna Blbulyan, Mayramik Sahakyan, Anait Vassilian, Karen Trchounian, Anna Poladyan

**Affiliations:** 1 Department of Biochemistry, Microbiology, and Biotechnology, Biology Faculty, YSU, Yerevan, Armenia; 2 Research Institute of Biology, Biology Faculty, YSU, Yerevan, Armenia

**Keywords:** l-Amino acids, *Ralstonia eutropha* H16, O_2_-tolerant Hydrogenases, H_2_-oxidizing Hydrogenase activity, Biocatalysts

## Abstract

*Ralstonia eutropha* H16 is a chemolithoautotrophic bacterium with O_2_-tolerant hydrogenase (Hyds) enzymes. Hyds are expressed in the presence of gas mixtures (H_2_, O_2_, CO_2_) or under energy limitation and stress conditions. O_2_-tolerant Hyds are promising candidates as anode biocatalysts in enzymatic fuel cells (EFCs). Supplementation of 0.5% (w/v) yeast extract to the fructose-nitrogen (FN) growth medium enhanced H_2_-oxidizing Hyd activity ~ sixfold. Our study aimed to identify key metabolites (l-amino acids (l-AAs) and vitamins) in yeast extract that are necessary for the increased synthesis and activity of Hyds. A decrease in pH and a reduction in ORP (from + 240 ± 5 mV to − 180 mV ± 10 mV values) after 24 h of growth in the presence of AAs were observed. Compared to the FN-medium control, supplementation of 7.0 μmol/ml of the l-AA mixture stimulated the growth of bacteria ~ 1.9 to 2.9 fold, after 72 h. The whole cells’ H_2_-oxidizing Hyd activity was not observed in control samples, whereas the addition of l-AAs, mainly glycine resulted in a maximum of ~ 22 ± 0.5 and 15 ± 0.3 U, g CDW^−1^ activity after 24 h and 72 h, respectively. Our results suggest a correlation between ORP, pH, and function of Hyds in *R. eutropha* H16 in the presence of key l-AAs. l-AAs used in small amounts can be proposed as signaling molecules or key components of Hyd maturation. These results are important for the optimization of O_2_-tolerant Hyds production as anode biocatalysts.

## Keypoints

1. l-AAs supplementation enhanced the biomass of R. eutropha H16.
2. The presence of l-AA during bacterial growth caused a decrease in ORP and pH.
3. l-AA is suggested to promote Hyds synthesis and enzymatic activity by reducing ORP and pH.

## Introduction

Nowadays, one of the main problems in our society is the energy crisis (Ripple et al. 2020). Therefore, finding alternative energy sources and developing methods and new strategies for accessing bioenergy is very important. *Ralstonia eutropha* H16 (*Cupriavidus* *necator* H16), a non-pathogenic, facultative chemolithoautotrophic β-proteobacterium, is subject to considerable biotechnological interest due to its capability of producing various metabolites, as well as bioplastics both heterotrophically and autotrophically (Pohlmann, et al. 2006; Cramm 2009; Grunwald et al. 2014; Raberg et al. 2018). It also synthesizes O_2_-tolerant [NiFe]-hydrogenases (Hyds) during lithoautotrophic growth on H_2_, CO_2,_ and O_2_: SH—a cytoplasmic NAD^+^—linked soluble Hyd, MBH—a membrane-bound Hyd, an AH—actinobacterial type Hyd, and RH—a regulatory Hyd (Goris et al. 2011; Schäfer et al. 2013; Lenz et al. 2018; Reeve et al 2022; Jugder et al. 2015, 2016). MBH and SH are attractive targets (bio-catalysts) for potential application in bio-electrochemical fuel cells (FCs): by oxidizing H_2_ into 2H^+^ and 2e^−^ they participate in electricity generation (Vincent et al. 2006; Poulpiquet et al. 2014). In this bacterium, O_2_-tolerant Hyd enzymes are expressed not only during lithoautotrophic growth (presence of potentially explosive gas mixtures) but also under stressful/energy-limiting conditions (Goris et al. 2011; Lenz et al. 2018; Poladyan et. al. 2019).

The heterotrophic growth of *R. eutropha* is supported by different carbon and energy sources, such as the tricarboxylic acid cycle’s intermediates, fatty-, sugar- and amino- acids, aromatic compounds, and alcohols (Pohlmann et al. 2006; Cramm 2009; Grunwald et al. 2014). Growth of the bacterium in a glycerol medium occurs slowly, but it has been shown that glycerol leads to catabolic derepression of Hyd enzyme synthesis (Jugder, et al. 2015). It was also demonstrated that the growth of *R. eutropha* H16 on lignocellulosic hydrolysate of brewery spent grains enhanced the activities of the MBH and SH enzymes (Poladyan et. al. 2019). Since Hyd synthesis can also be stimulated during the heterotrophic growth of *R. eutropha*, it would be beneficial to identify new conditions under which increasing Hyd enzyme synthesis and/ or activity occurs.

We recently serendipitously observed that supplementation of a small amount (0.5%) of Luria–Bertani (LB) medium to a minimal fructose-nitrogen (FN) medium led to increased Hyd enzyme activity of *R. eutropha*. The components of LB medium are yeast extract (YE) and tryptone: the YE is a complex of amino acids, carbon, sulfur, trace nutrients, vitamin B complex, and other growth factors essential for diverse microorganisms (Li et al. 2011; Hakobyan et al. 2012); the main components of tryptone are l-amino acids. Some component(s) of YE or tryptone might be essential for the increased synthesis and activity of the Hyd in *R. eutropha*. This study aimed to identify this component and determine how it might impact Hyd activity.l-amino acids have an essential role in bacterial growth and metabolism: they serve as building blocks for protein synthesis but are also known as signaling molecules for different pathways of prokaryotic and eukaryotic cells (Chantranupong et al. 2015). Different receptors recognize the extracellular AAs and their derivatives, for example, G protein-coupled receptors, eukaryotic ligand-gated ion channels, and microbial chemoreceptors (Parkinson et al. 2015; Ortega et al. 2017). Amino acids utilization by bacteria depends on their genetic differences and nutritional availability from the environment, this leads to phenotypes significant variations even when they belong to the same species, even in a recent study, the utilization of single AAs was considered for categorization of bacteria (Liu et al. 2020). The study by Liu et al. (2020) determined the single AA utilization profiles of seven bacterial species and demonstrated that most bacteria have species-specific patterns of amino acid consumption.

It is known, that proline is involved in various cellular processes and is critical for cell growth and protein synthesis. Proline participates in osmoregulation, involves in redox signaling, provides resistance to stress, and is used by organisms for the secondary metabolites’ biosynthesis (Christgen 2017). Another amino acid glycine can lead to both stimulation and inhibition of bacterial growth depending on the type of bacteria and concentration (Kajikawa et al. 2002; Minami et al. 2004; Gabrielyan and Trchounian 2012). Also was shown, that some of the l-Amino acids are essential for [FeFe]-Hyds maturation (Kleinhaus et al. 2021).

The study aimed to reveal the components (single l-AAs, vitamins) in yeast extract responsible for the induction of H_2_-oxidizing activity during the heterotrophic growth of *R. eutropha* H16, which proved to involve mainly glycine and proline.

## Materials and methods

### Cultivation conditions and growth characteristics of bacteria

*R. eutropha* H16 (DSM 428) was kindly provided by Dr. Oliver Lenz (Technical University Berlin, Berlin, Germany). *R. eutropha* was grown under heterotrophic conditions, using FN (Fructose-Nitrogen) minimal mineral solution (Lenz et al. 2018; Poladyan et al. 2019). Where indicated, 7.0 μmol/ml l-amino acids (l-AA) and B group of vitamins were added (Hakobyan et al. 2012). The FN solution was a composition of 10 × H16 buffer (100 ml), 850 ml H_2_O, as well as 20% w/v of 10 mL NH_4_Cl, 20% w/v of 1 mL MgSO_4_ × 7H_2_O, 1% w/v of 1 mL CaCl_2_ × 2H_2_O, 0.001% w/v of 1 mL NiCl_2_, 0.5% w/v of 1 mL FeCl_3_ × 6H_2_O, and 40% w/v of l10 mL fructose. The 10 × H16 buffer was composed of 15 g KH_2_PO_4_ and 90 g Na_2_HPO_4_ × 12 H_2_O, and 1 L H_2_O, pH 7.0. Bacterial aerobic growth was achieved on a shaker at 130 rpm, 30 °C. Aerobic condition for cultivation experiments was achieved by using 250 mL baffled flasks with 100 mL FN. The L-AA and B group vitamins used included Gly—l-glycine, Ala- l-alanine, Asn- l-asparagine, Asp- l-asparagine acid, Pro— l-proline, Tyr— l-tyrosine, Cys— l-cysteine, Ser— l-serine, Glu— l-glutamic acid, Arg— l-Arginine, His— l-Histidine and B1—Thiamine, B3—Nicotinic acid, B6 -Pyridoxine. 1.5% of a bacterial pre-culture (starter) was used to inoculate cultures and cultivation was carried out at 30 °C for 72 h.

Occasionally, LB (Luria–Bertani) (0.5% w/v) or individual LB components were added to the cultures. The composition of the LB liquid medium was tryptone 10 g/L, YE 5 g/L, and NaCl 10 g/L. For inoculation (starters), bacteria were grown under the conditions described above, 37 °C (Schwartz et al. 2009; Jugder et al. 2015, 2016; Lenz et al. 2018).

Bacterial growth was determined by a Cary 60 UV–vis spectrophotometer (Agilent, USA). µ, the specific growth rate, calculated as OD’s ln2/doubling time (logarithmic growth phase) and expressed as h^−1^(Gabrielyan et al. 2010). CDW, g L^−1^, the bacterial cell dry weight, was determined and applied to evaluate the yield of bacteria. The medium’s pH was measured with the pH electrode (HJ1131B, Hanna Instruments, Portugal): mediums pH was controlled with solutions of HCl (0.1 N) and NaOH (0.1 M).

### Oxidation and reduction potential measurement

The medium’s oxidation and reduction potential (ORP) was measured by using two electrodes, namely an oxidation–reduction platinum, Pt, EPB-1, and a titanium-silicate Ti-Si, EO-02, Measuring Instruments Enterprise, Gomel, Belarus, electrodes. The Ti-Si electrode having no sensitivity to O_2_ or H_2_ was used to determine the overall ORP of the microbial cultures. Pt electrode senses the O_2_ and H_2_. Electrodes were checked in a solution, with a composition of 0.049 M K_3_[Fe(CN)_6_] and 0.05 M K_4_[Fe(CN)_6_]*3H_2_O, pH 6.86: the readings for both redox electrodes at 25 °C were + 250 ± 5 mV (Vassilian and Trchounian 2009; Poladyan et al. 2019; Usmani et al. 2022).

### Obtaining cells extracts and monitoring of Hyd activity

Bacterial growth was monitored by determining the optical density, OD_600_, under 600 nm. Cells were harvested every 24 h during a 72 h period under 5000 rpm, 4 °C, 10 min centrifugation. Collected bacteria were washed with K-PO_4_ buffer, 50 mM K-PO_4_, pH 7.0.

The whole cells’ H_2_-oxidizing total activity was monitored by methylene blue (MB) reduction, 570 nm, 30 °C, using a spectrophotometer, Cary 60 UV–vis, Agilent Technologies, USA. 15 to 20 mL of bacterial whole cells was supplemented into a 1.9 mL reaction mixture of 50 mM K_i_PO_4_ buffer, pH 7.0, and H_2_-saturated. MB was provided as the acceptor of artificial electrons (Lenz et al. 2018).

For determination of MBH and SH activities the cell’s crude extracts were obtained using Sonifier Branson SFX150, Ultrasonic Cell Disruptor 4C15, Mexico, as described (Karapetyan et al. 2020): the cells (1–2 g wet weight) were disrupted by sonication, 30 W power, 2.5 min with 0.5 s pulses. Cell debris and membranes were separated from the soluble protein fraction by ultracentrifugation, 36000xg at 4 °C for 45 min (Preparative Ultracentrifuge Bechman/Spinco L2, Fixed-Angle Rotor Type 50 Titanium, Beckman Instruments GmbH, Munchen, Germany). The supernatant with soluble protein fraction was used for measurements of SH-, whereas the pellet (membrane) for the MBH- activities (Lenz et al. 2018; Poladyan et. al. 2019).

The SH was measured in anaerobic cuvettes using NAD^+^ as an acceptor of electrons under 365 nm, with a Cary 60 UV–vis spectrophotometer, Agilent Technologies, USA. SH-containing soluble protein fractions (5 to 10 μL) were added to a 1.9 mL reaction mixture having 1 mM NAD^+^ and 50 mM H_2_-saturated Tris/HCl buffer, pH 8.0. The unit of Hyd activity was defined as the sum of enzyme which catalyzes the transformation of 1 μmol of H_2_ per min/mg of protein (Schwartz et al. 2009; Jugder et al. 2015; Lenz et al. 2018).

One unit (U) of Hyd enzyme activity corresponds to 1 µM H_2_ oxidized per min and 1 mg protein (Lenz et al. 2018) or g CDW.

Protein concentration was measured by the Lowery method (Waterborg and Matthews 1984).

### Reagents used in the research and data processing

Data were processed using Microsoft Excel 2016. Data was determined from 5 independent experiments; standard errors were calculated by the function of Microsoft Excel, 2016. To check the difference in mean records between measurement series the P (Student’s criteria) was measured (Poladyan et al. 2018; Trchounian et al. 2017). Upon P < 0.05, the difference is considered as significant. Analytical purity reagents (l-glycine, l-proline, fructose, etc., Carl Roth GmbH, Germany) were used in the experiments.

## Results

### ***Effect of LB medium components on microbial H***_***2***_***-oxidising activity, growth, and ORP kinetics***

**Fig. 1:**
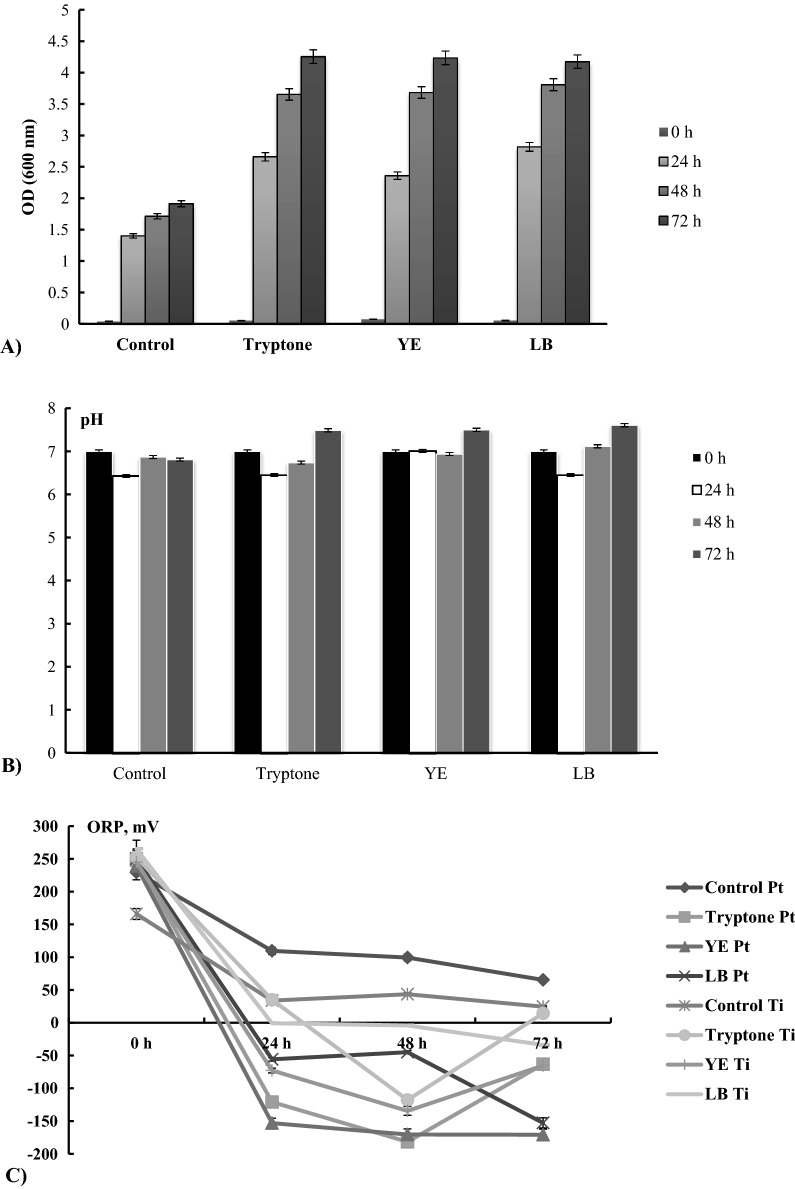
Effects of LB medium components on *R. eutropha* H16 bacterial growth parameters. Bacteria were grown aerobically, pH 7.0, for 72 h: Kinetic of **A** OD (600 nm) (optical density), **B** pH, and C) ORP, (n = 5, p < 0.05). The ORP was measured by using Pt and Ti-Si electrodes and was expressed in mV [vs Ag/AgCl (saturated by KCl)]. Control is FN (Fructose-Nitrogen medium), 0.5% of LB (Luria–Bertani Medium), and YE- (Yeast extract) and tryptone were supplemented into FN. The average data of 3 independent measurements are presented with standard errors

The pH, ORP, and growth of *R. eutropha* H16 were monitored under supplementation of the growth medium with 0.5% LB medium, or 0.5% yeast extract (YE), and 0.5% tryptone alone. As was mentioned before (Lenz et al. 2015; 2018) during heterotrophic growth Hyd activity is mainly observed in starved cells, consequently, in this study, we followed bacterial growth up to the late stationary phase. Bacterial growth on the FN medium without the additional components listed above was considered a control experiment. As mentioned above *R. eutropha* H16 synthesizes four different Hyd enzymes and therefore total Hyd activity was determined. Both LB medium and its components, when added to the FN medium, stimulated bacterial growth compared to the control (Fig. 1A). After 72 h of growth, improvements were near two-fold for each addition (Fig. 1A), by providing vitamins, carbon and nitrogen sources.

**Fig. 2:**
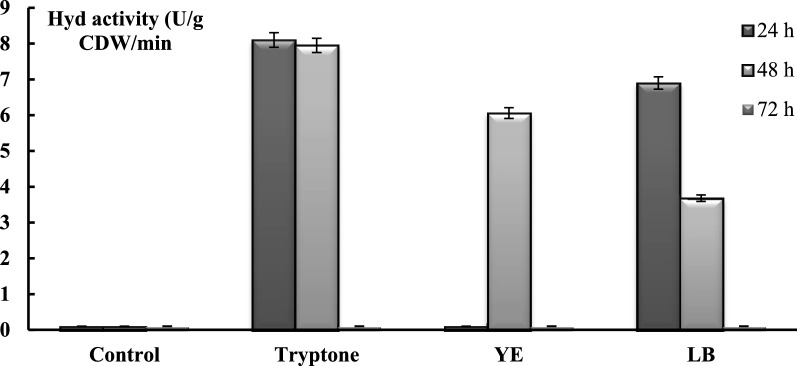
LB medium components influence H_2_-oxidizing total Hyd activity of *R. eutropha* H16 whole cells. CDW is Cell Dry Weight. One unit of Hyd activity was defined as the amount of enzyme which that catalyzes the conversion of 1 mmol of H_2_ per min. For the other details, see Materials and methods and the legends in Fig. 1, (n = 5, p < 0.05)

During bacterial growth, no significant change in the medium’s pH was observed (Fig. 1B). In contrast to the control, a decrease in ORP from positive to negative values was recorded for both the Pt (+ 110 ± 10 mV to—20 ± 10 mV) and Ti-Si (+ 40 ± 10 mV to − 120 ± 10 mV) electrodes after 24 h of growth (Fig. 1C). Correlating with the observed decrease in ORP in the presence of LB components (tryptone, YE), it was also noted that the total H_2_-oxidising activity in whole cells increased significantly (Fig. 2). After 24 h of growth activity of ~ 8 ± 0.06 U g CDW^−1^ and 7 ± 0.02 U g CDW^−1^ Hyd activity was determined after supplementation with tryptone and LB, respectively. H_2_-oxidizing Hyd activity was also recorded in the presence of tryptone and high activity was maintained up to 48 h of growth, while in the case of LB, it decreased by approximately 50% (equal to 3.60 ± 0.05 U g (CDW)^−1^) (Fig. 2). The total H_2_-oxidizing Hyd activity in the presence of YE was 6 ± 0.06 U after 48 h of growth (Fig. 2). After 72 h, Hyd activity was negligible, suggesting that the decrease of Hyd activity correlated with the microbial late stationary growth phase (Figs. 1A, 2).

### ***The effect of L-AAs and group B vitamins on bacterial whole cells’ H***_***2***_*** -oxidizing Hyd activity***

Both tryptone and YE include amino acids and vitamins, to determine whether these components were responsible for the increased total H_2_-oxidizing activity, the growth of *R. eutropha* H16 was followed in the FN medium with 7.0 μmol/ml of the l-AA (Pro, Glu, Asn, Asp, Tyr, His, Arg, Ser, Cys, Ala, and Gly) and vitamins (Thiamine, Nicotinic acid, Pyridoxine) supplementation. Bacterial growth on FN standard medium without l-AAs and vitamin supplementation were considered as a control experiment.

**Fig. 3:**
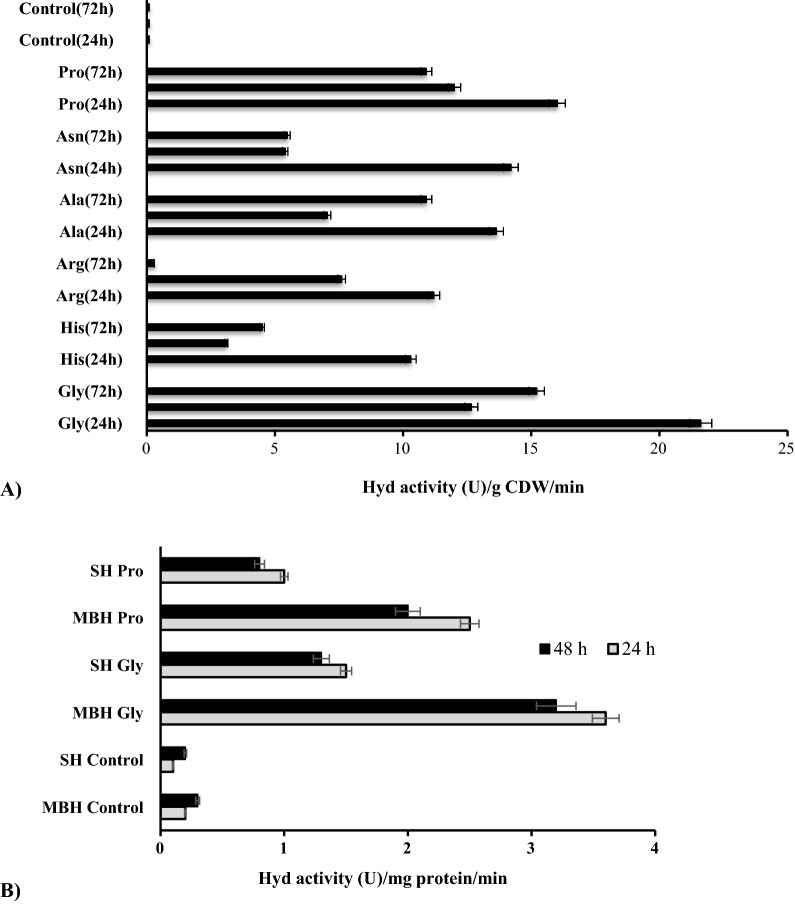
H_2_-oxidizing total Hyd activity of whole cells (**A**) and SH and MBH activities (**B**) of *R. eutropha* H16 under influence of L-AAs. L-AAs (Gly, Ala, Asn, Pro, Arg, His) were added in FN medium at 7.0 μmol/ml concentration. CDW is Cell Dry Weight. Gly- Glycine, Ala- Alanine, Asn- Asparagine, Pro- Proline, Asp-asparagine acid, Tyr -tyrosine, Cys -cysteine, Ser -serine, Glu -glutamic acid, Arg -Arginine, His -Histidine and B1-Thiamine, B3-Nicotinic acid, B6-Pyridoxine. SH and MBH activities were determined in Pro- and Gly—supplemented samples. For the other details, see Materials and methods and the legends in Fig. 3 (72 h, n = 5, p < 0.05)

*R. eutropha* whole cells H_2_-oxidizing Hyd activity was determined during 72 h of growth upon supplementation of L-AAs and vitamins. Note, that the H_2_-oxidizing Hyd activity was absent in control samples, while it was recorded in the samples, where Gly, Ala, Asn, Arg, His, and Pro were added (Fig. 3).

Our experiments show that in the case of Asp, Glu, Tyr, Cys, Ser l-AAs, and group B vitamins addition H_2_-oxidizing Hyd activity was negligible during the entire time of experimental measurements. The H_2_-oxidizing Hyd activity was recorded starting from 24 h of *R. eutropha* growth in the presence of Gly, Ala, Pro, Arg, His, and Asn L-AAs, at the same time, the negative ORP values of the mediums were shown (Fig. 5). In Pro, Ala, Asn, and Arg, His containing samples the H_2_-oxidizing Hyd activities were ~ 14 ± 0.5 U g CDW^−1^, and 11 ± 0.5 U g CDW^−1^, respectively (Fig. 3). The maximal ~ 22 ± 0.05 U (g CDW)^−1^ H_2_-oxidizing Hyd activity was determined in Gly containing samples after 24 h of *R. eutropha* H16 growth (Fig. 3). Consequently, cell extract with Gly and Pro supplemented samples were prepared, in which the MBH and SH activities were determined and presented in Fig. 3B: as described for whole cells, compared to the control, Hyd activities are significantly stimulated. Maximal Hyd activity occurred at 24 h of bacterial growth. In soluble extracts of cells grown with Gly or Pro supplementation, the SH activity was 1.50 ± 0.02 and 1.00 ± 0.01 U/mg protein, respectively (Fig. 3B). In the case of MBH activity, membrane extracts of Gly- and Pro-grown cells showed ~ 2.5-fold higher activity, up to 3.6 ± 0.03 and 3 ± 0.02 U/mg protein, respectively, compared with SH activity.

### The effects of l-AAs and group B vitamins on R. eutropha H16 growth properties

**Fig. 4:**
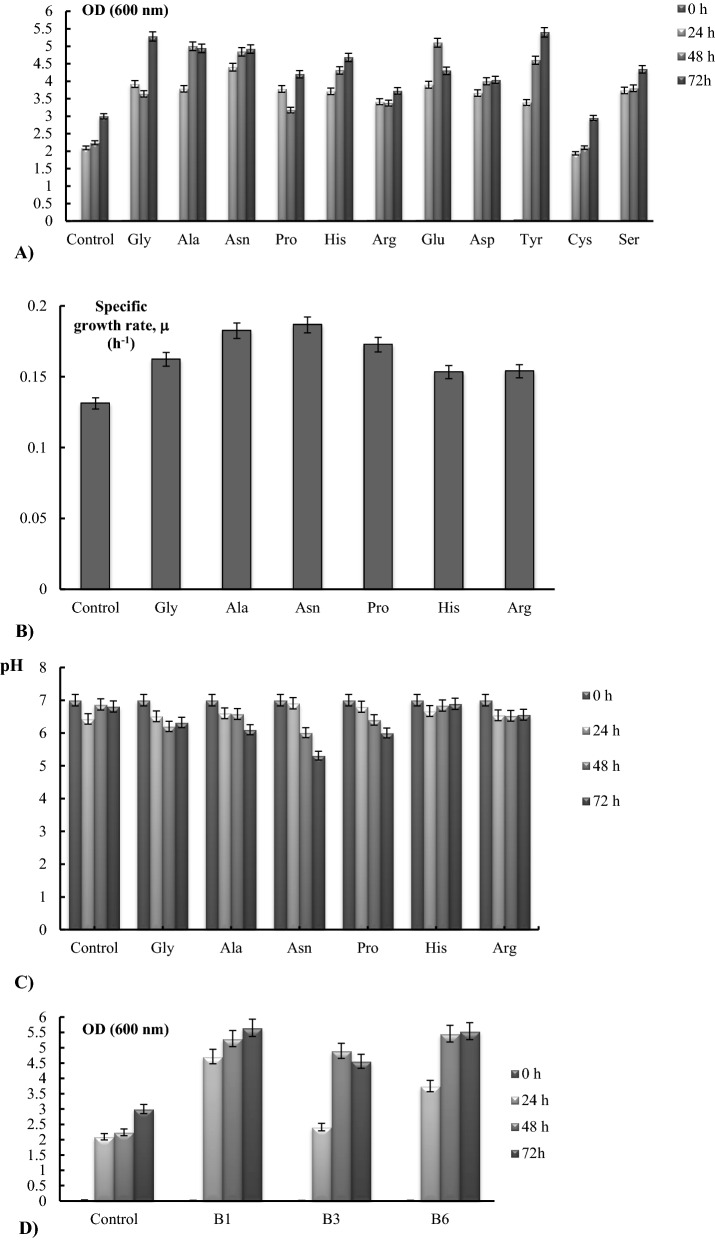
*R. eutropha* H16 growth in the presence of l-Amino acids (AAs). Bacteria were grown aerobically, at pH 7.0, for 72 h: Kinetic of **a** OD (600 nm) with the addition of L-AAs; **b** specific growth rate, μ (h^−1^) and **c** pH; **d** bacterial growth (OD) with the addition of vitamins; n = 5; p < 0.05. Control is FN (without AAs addition), L-AAs and vitamins were added in FN medium at 7.0 μmol/ml concentration. The average data of 4 independent measurements are presented with standard errors

The effect of L-AA on the stimulation of bacterial growth was observed after 24 h of L-AAs supplementation, but the maximum growth stimulation was recorded after 72 h. Compared to the control, the supplementation of Arg, His, Pro, Asn, Ala, and Gly stimulated *R. eutropha* H16 bacterial growth ~ 1.2, 1.5, 1.9, 2.3, 2.7, and 2.9 fold, respectively (Fig. 4A).

The specific growth rate (μ) of the samples with AAs was also stimulated ~ 1.2 to 1.4 fold (Fig. 4B). Although, the highest bacterial biomass was recorded in the presence of Gly (Fig. 4A), upon Asn supplementation the μ of bacteria was maximal, ~ 0.2 ± 0.03/h (Fig. 4B). Note, group of B vitamins (Thiamine, Nicotinic acid, Pyridoxine) and Asp, Glu, Cys, and Tyr, supplementation also stimulated bacterial growth after 72 h ~ 2 and ~ 1.5 fold, respectively (Fig. 4A, D).

As in the case of tryptone and YE, the pH of bacterial growth mediums with the Gly, Ala, His, Arg, Asn, and Pro L-AAs containing samples was significantly decreased (Fig. 4C). However, in the presence of Asp, Glu, Cys, Tyr, and Group B vitamins only slightly acidification of the pH was recorded.

**Fig. 5:**
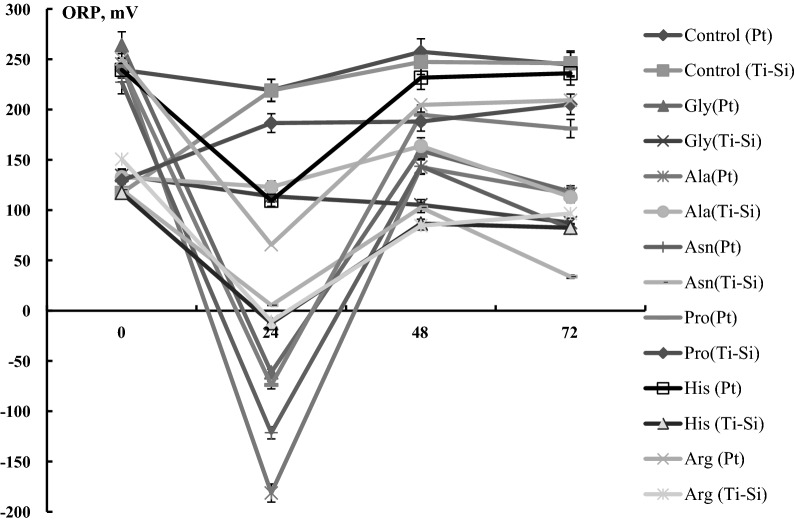
L-AAs influence on the kinetics of ORP of *R. eutropha* H16. Bacteria were grown aerobically, at pH 7.0, for 72 h, n = 5, p < 0.05. The ORP was measured by using Pt and Ti-Si electrodes. For the other details, see Materials and methods and the legends in Figs. 1, 3

Kinetics of ORP, recorded by redox electrodes (Pt and Ti-Si) are presented in Fig. 5. The initial ORP values of the samples were + 120 ± 15 mV (Ti-Si) and + 250 ± 10 mV (Pt)*.* Compared to the control experiment, the supplementation of AAs leads to an ORP decrease of up to -180 ± 5 mV values*.* The decrease of ORP to − 70 ± 5 mV, − 120 ± 10 mV, and − 180 ± 10 mV were shown upon the addition of Gly, Pro, and Asn, Ala, respectively, after 24 h. Moreover, after 48 h the ORP values of the Pt electrode recovered to positive and remained almost unchanged in 72 h of bacterial growth.

## Discussion

The synthesis and activity of *R. eutropha* H16 Hyd enzymes are significantly dependent on environmental and experimental conditions (Goris et al. 2011; Lenz et al. 2015). As *Knallgas* bacteria, Hyds are synthesized during autotrophic aerobic growth using H_2_ and CO_2_, where RH (regulatory hydrogenase) is responsible for the initiation of Hyd synthesis. However, the mechanisms of initiation of Hyd synthesis and activity of Hyds upon heterotrophic growth are uncertain. Moreover, it is challenging during heterotrophic growth to design the conditions for enhanced biomass production and Hyd synthesis and activities (Grunwald et al. 2014; Lenz et al. 2018; Poladyan et al. 2019).

**Fig. 6:**
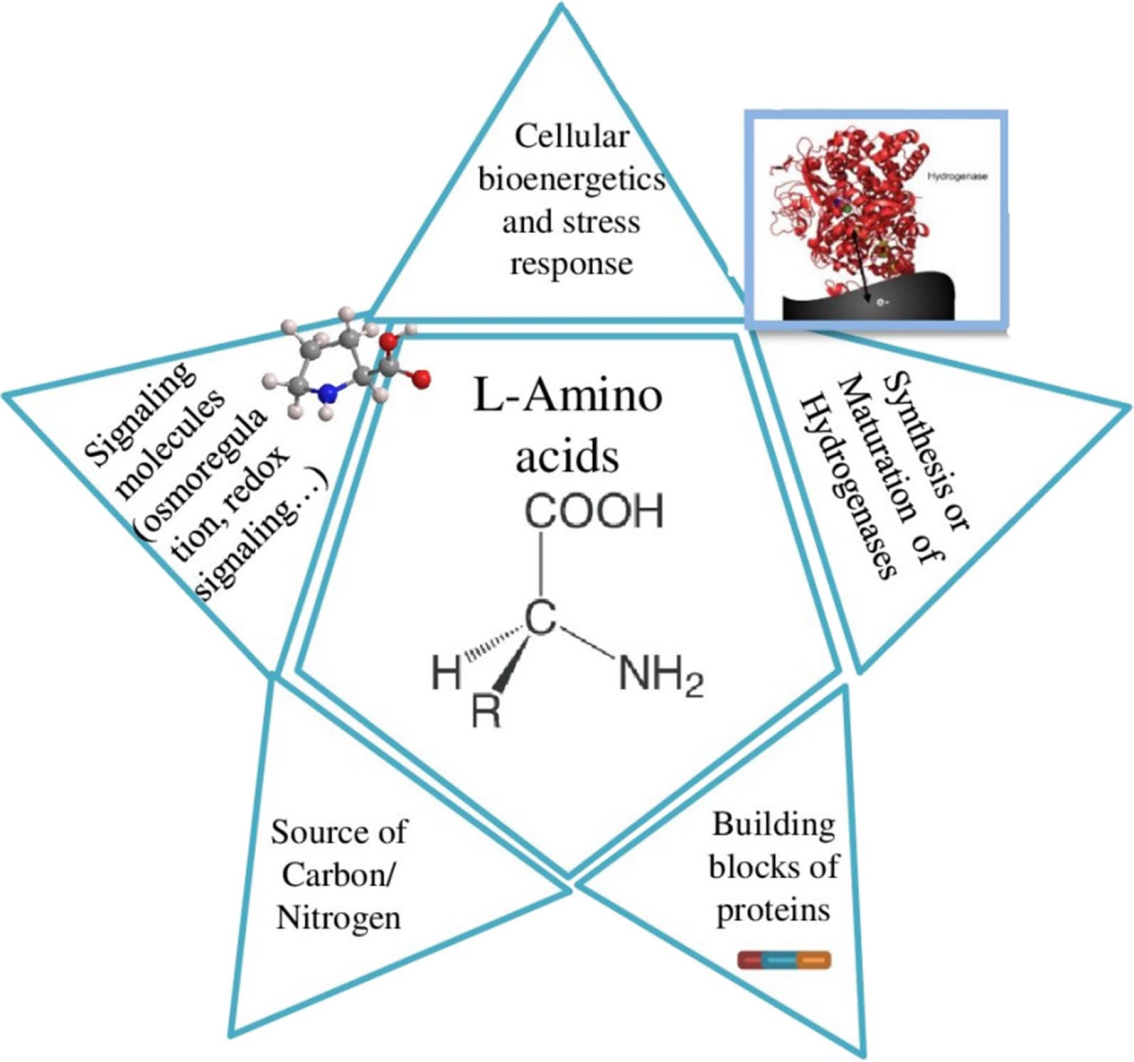
Schematic presentation of the effects of l-Amino acids on the metabolism of bacteria

The current research indicates that the addition of amino acids (AAs) improves the growth and biomass production of *R. eutropha* H16 and promotes O_2_-tolerant Hyds synthesis and activity during bacterial heterotrophic growth. The possible effects of l-AAs on the metabolism and Hyd activation of bacteria, in general, are considered in Fig. 6 (Christgen 2017, Christgen and Becker 2019; Lenz et al. 2018; Sánchez-Andrea 2020; Kleinhaus et al. 2021).

Bacterial growth was accompanied by slight acidification of the medium. The recorded decrease in ORP also supports the contention that improved growth resulted in increased Hyd enzyme activity. An increase in pH late in growth correlated with a decrease of H_2_-oxidising total Hyd activity, e.g. after 72 h of bacterial growth, especially with LB addition.

It is well known, that the ORP (*E*_h_) of bacterial liquid culture is affected both by the ratio of oxidized and reduced fermentation products and pH according to the equation (*E*_h_ = *E*_0_ + (RT/nF) ln ([ox]/[red]) + (RT/nF) ln [H^+^]) with a decrease of E_h_, pH rises (Poladyan et al. 2013). However, there is a poorly understood relationship between the observed decrease in *E*_h_ and pH of bacterial growth mediums, but during fermentation, many complex redox processes occur that cannot be explained by the equation. ORP values can be affected by the activity of many membrane-associated oxidoreductases involved in the cellular proton cycle, as well as H_2_-oxidizing and H_2_-evolving Hyds, which are one of these enzymes (Bagramyan et al. 2000; Lukey et al. 2010; Trchounian et al. 2012; Poladyan et al. 2013). Thus, ORP is probably reduced as a consequence of the induction of Hyd activity.

The stimulating effects of l-AA likely are connected to the redox status of the cell (e.g. NAD^+^/NADH), which possibly leads to the activation of gene expression (Fig. 6). Alternatively, but less likely, the l-amino acids might be essential for optimal Hyd biosynthesis or enzyme activation (Fig. 6) (Kleinhaus et al. 2021). They might be involved in various metabolic pathways such as the Krebs cycle and promote the synthesis of redox equivalents such as NADH, which plays a significant role in activating Hyds, specially NAD^+^/NADH-dependent SH activity.

Different bacteria may have different abilities to use AAs depending on their genetic and host differences, as well as the availability of nutrients from the environment, leading to significant differences in their phenotypes even if they belong to the same species (Liu et al. 2020). Among the tested AAs, the maximal H_2_-oxidizing Hyd activity of whole cells was observed during Gly supplementation (Fig. 3A). Compared to SH, MBH activity was stimulated (Fig. 3B) after 24 h of growth. Gly is often used as a main organic N source in field research because of its abundance in many soils; it can be utilized rapidly by microorganisms (Xue et al. 2022). The observation may be related to the properties of Gly and the characteristics of the bacteria that use it. The preference for Gly within the soil microbial community was shown by ^13^C glycine-uptake to be more rapidly incorporated into phospholipid during the first 6 h, and increased nitrogen from glycine was also found in cells (Andresen et al. 2014).

It was also shown that glycine and l-threonine were the only ‘energy amino acids’ that enhanced the specific growth rate of *E. coli*, when supplemented solely to a minimal medium with glucose as the carbon source (Han et al. 2002). The effect of Gly was concentration-dependent and 30 mmol/L Gly decreased the growth, changed the cell morphology from rod to spheroid, and degradation of intracellular proteins occurred. In contrast, a lower amount of glycine initially improved the growth of *E. coli* (Han et al. 2002; Gabrielyan and Trchounian 2012). According to the literature, in bacterial cells, Gly mainly can be metabolized into acetyl-phosphate, with further conversion to pyruvate and acetyl-CoA, or conversely, glycine can be metabolized to serine (Sánchez-Andrea et al. 2022). Recent studies identified that Gly and pyruvate, or intermediates of their metabolism, might serve as possible H-cluster’s building blocks, the active site of [FeFe] Hyds (Kleinhaus et al. 2021).

Due to its osmolytic properties, Pro provides cellular protection against abiotic stress such as osmotic shock. Proline is used as a source of nitrogen and carbon during oxidative metabolism and supports growth and energy requirements (Christgen 2017; Christgen and Becker 2019). Proper management of proline levels helps maintain protein biosynthesis in response to stress and energy needs (Christgen 2017; Christgen and Becker 2019). Therefore, future studies will be necessary to determine if proline acts by inducing Hyd synthesis in *R. eutropha* H16, possibly by acting in stress response systems (Fig. 6).

In contrast to Asp, the addition of Asn into *R. eutropha* H16 culture, the H_2_-oxidizing total Hyd activity was induced, hence Asn can be offered as the supplemental source of the NH(CH_2_)_2_ moiety for Hyds (Kleinhaus et al. 2021). Considering the histidine effect, *R. eutropha* H16 has an H_2_-sensitive pathway responsible for H_2_-dependent expression of the Hyd genes is controlled by a gene, which includes a histidine protein kinase that controls the activity of a response regulator to control Hyd gene (Lenz et al. 2002; 2018). Whether this might be linked to a response to histidine addition is unclear.

As was mentioned above, during heterotrophic growth Hyds of *Rasltonia* H16 are active in starved (energy limitation) cells: microbial starvation followed by cell lysis usually leads to the environmental availability of free AAs (Ochi 2017). So, small amounts of AAs in the medium might mimic “starvation”, which possibly will turn on the cell starvation-survival-associated response, leading also Hyd enzymatic activation. Future studies are required to understand the detailed mechanism of influence of single AAs on the growth and Hyd activity of *R. euotrpha* H16.

The combination of different AAs and carbon sources had different effects on growth properties and H_2_ production (activity of Hyd enzyme) by *Rhodobacter sphaeroides* strains (Gabrielyan et al. 2010, Gabrielyan and Trchounian 2012). The differential effect of AAs on bacterial growth and Hyd activity probably can be connected with the transport and metabolic pathways of AAs. Diffusion of AAs through the plasma membrane does not occur (Burkovski and Krämer 2002; Moe 2013). Bacterial take up AAs by transporters that use the electrochemical ion potential across the plasma membrane (secondary transport systems) or via primary active transport using ABC transporters (Saier and Milton 2000).

Recently was demonstrated the successful engineering and optimization of the synthetic reductive glycine pathway in  *C. necator (R. eutropha)*, replaced the Calvin cycle for supporting growth on formate (Claassens et al. 2020).

Thus, our results suggest a correlation between improved heterotrophic growth and increased activity of Hyds in *R. eutropha* H16. Hence, l-AAs used in small amounts can be proposed to improve growth and improve and prolong the synthesis of O_2_-tolerant Hyds, which can be considered anodic catalysts in biological fuel cells.

## Data Availability

All data generated or analyzed during this study are included in this published manuscript.

## Abbreviations

AH: Actinobacterial-type Hyd
Ala: l-Alanine
Asn: l-Asparagine
Asp: l-Asparaginic acid
CDW: Cell dry weight
Cys: l-Cysteine
EFCs: Enzymatic fuel cells
Glu: l-Glutamic acid
Gly: l-Glycine
Hyds: Hydrogenases
L-AAs: l-Amino acids
LB: Luria–Bertani
MB: Methylene blue
MBH: Membrane-bound Hyd
ORP: Oxidation–reduction potential
Pro: l-Proline
RH: Regulatory Hyd
Ser: l-Serine
SH: Soluble Hyd
Tyr: l-Tyrosine
